# Prokineticin 2 is a catabolic regulator of osteoarthritic cartilage destruction in mouse

**DOI:** 10.1186/s13075-023-03206-4

**Published:** 2023-12-06

**Authors:** Jiye Yang, Youngnim Shin, Hwee-Jin Kim, Hyo-Eun Kim, Jang-Soo Chun

**Affiliations:** National Creative Research Initiatives Center for Osteoarthritis Pathogenesis and School of Life Sciences, Gwangju Institute of Science and Technology, Gwangju, 61005 Republic of Korea

**Keywords:** Prokineticin, Chondrocytes, Osteoarthritis, Matrix-degrading enzymes, Mechanical allodynia, Biomarker

## Abstract

**Background:**

Our preliminary study indicates that the multi-functional protein, prokineticin 2 (Prok2), is upregulated in osteoarthritic (OA) chondrocytes as a target of the hypoxia-inducible factor (HIF)-2α. This study aims to elucidate the potential roles of Prok2 in OA.

**Methods:**

Prok2 expression was assessed through microarray analysis in chondrocytes and confirmed via immunostaining in OA cartilage. Experimental OA was induced through destabilization of the medial meniscus (DMM). Functions of Prok2 were assessed by adenoviral overexpression, intra-articular (IA) injection of recombinant Prok2 (rProk2), and knockdown of Prok2 in joint tissues. We also explored the potential utility of Prok2 as an OA biomarker using enzyme-linked immunosorbent assay (ELISA).

**Results:**

HIF-2α upregulated Prok2, one of the prokineticin signaling components, in OA chondrocytes of mice and humans. Adenoviral overexpression of Prok2 in chondrocytes and cartilage explants, as well as the application of rProk2, led to an upregulation of matrix metalloproteinase (MMP)3 and MMP13. Consistently, the overexpression of Prok2 in joint tissues or IA injection of rProk2 exacerbated cartilage destruction and hindpaw mechanical allodynia induced by DMM. However, the knockdown of Prok2 in joint tissues did not significantly affect DMM-induced cartilage destruction. Additionally, despite being a secreted protein, the serum levels of Prok2 in OA mice and human OA patients were found to be below the range detected by ELISA.

**Conclusion:**

The upregulation of Prok2 exacerbates OA cartilage destruction and hindpaw mechanical allodynia. However, its knockdown is not sufficient to inhibit experimental OA and Prok2 is not a potential candidate serum biomarker of OA.

**Supplementary Information:**

The online version contains supplementary material available at 10.1186/s13075-023-03206-4.

## Background

Osteoarthritis (OA) can develop due to multiple risk factors, including mechanical stress, metabolic disorders, inflammaging, and more [1–3]. The disease is characterized as a whole-joint disorder, featuring cartilage destruction, synovial inflammation, osteophyte formation, subchondral bone remodeling, etc. [4]. Among these OA manifestations, a hallmark of OA is progressive articular cartilage destruction, primarily regulated by chondrocytes through the production of matrix-degrading enzymes and/or the downregulation of cartilage extracellular matrix (ECM) molecules [5, 6]. Among the various matrix-degrading enzymes, animal model-based studies have revealed that matrix metalloproteinase 3 (MMP3), MMP13, and ADAMTS5 play crucial roles in OA cartilage destruction [7–9].

The expression of matrix-degrading enzymes can be regulated by various extracellular factors, including the pro-inflammatory cytokine interleukin (IL)-1β [10]. In previous studies, we demonstrated that the transcription factor hypoxia-inducible factor (HIF)-2α plays a critical role in OA pathogenesis by regulating the expression of matrix-degrading enzymes [11]. Specifically, HIF-2α directly upregulates MMP3 and MMP13 as targets in chondrocytes [11]. Beyond this direct regulation, HIF-2α amplifies the expression of matrix-degrading enzymes in chondrocytes through downstream signaling. For instance, downstream targets of HIF-2α, such as IL-6 and NAMPT (visfatine), further induce the expression of MMP3 and MMP13 [12–14]. Additionally, HIF-2α upregulates the zinc importer ZIP8, leading to zinc influx and activation of the MTF1 transcription factor, which enhances the expression of MMP3 and MMP13 [15, 16]. Cholesterol hydroxylases CH25H and CYP7B1 are also targeted by HIF-2α in chondrocytes, and their oxysterol metabolites activate the transcription factor RORα, thereby upregulating matrix-degrading enzymes [17]. We identified numerous HIF-2α targets in chondrocytes that act as catabolic regulators of OA pathogenesis in mice, including lipopolysaccharide binding protein (LBP) and CD14 [18], RNA-binding protein ZFP36L1 [19], arginase II [20], among others. Consequently, HIF-2α appears to be a pivotal catabolic transcription factor that regulates multiple catabolic molecules in chondrocytes.

In preliminary experiments, we identified prokineticin 2 (Prok2) as a novel target of HIF-2α in chondrocytes through bioinformatic analysis of various microarray datasets obtained from OA-like chondrocytes. Prok2 belongs to a new family of chemokines; the prokineticin signaling system comprises two secreted proteins (Prok1 and Prok2) and two cognate G protein-coupled receptors (ProkR1 and ProkR2) that are widely expressed in many tissues and exhibit great versatility [21, 22]. The prokineticin system modulates numerous essential biological functions, including angiogenesis, neurogenesis, metabolism, circadian rhythms, hematopoiesis, immune response, and pain perception [23–26]. Among the prokineticin signaling components, Prok2 has been reported to play a role in autoimmune diseases, including psoriasis, rheumatoid arthritis (RA), and collagen-induced arthritis (CIA) [27–29]. Studies have demonstrated that CIA can be suppressed by the inhibition of Prok2 [28] or the blockade of prokineticin receptors [29]. The prokineticin system also appears to be associated with RA in humans [30]. However, the potential role of prokineticins in OA pathogenesis remains unknown. Here, we investigated whether the upregulated Prok2 in OA chondrocytes regulates OA pathogenesis. Our findings suggest that the upregulation of Prok2 functions as a catabolic regulator of OA cartilage destruction and OA-associated mechanical allodynia in mice. Nonetheless, knocking down Prok2 alone is not sufficient to inhibit experimental OA cartilage destruction. Additionally, Prok2 is undetectable in both OA mouse or human sera, indicating that it is not a viable candidate biomarker for OA.

## Methods

### Human OA samples

Human OA cartilage (*n*=5 patients) was obtained from individuals undergoing arthroplasty. The characteristics of the sampled individuals with OA from whom the samples were sourced have been previously described [31]. The Institutional Review Board of Wonkwang University Hospital approved the use of these materials, and all participants provided written informed consent before undergoing the operative procedure. Additionally, we used human sera obtained from the population-based Hallym Aging Study, a prospective cohort study investigating the health of the elderly community [32, 33]. Serum levels of Prok2 were measured in a convenience sample, consisting of randomly selected Kellgren-Lawrence (KL) grade 0 individuals without OA (18 females) and KL grade 3 or 4 OA patients (18 females).

### Experimental OA in mice

All mice were maintained under specific pathogen-free conditions, and all animal experiments were approved by the Gwangju Institute of Science and Technology Animal Care and Use Committee. Post-traumatic experimental OA was induced in 12-week-old male C57BL/6J mice by performing DMM (destabilization of the medial meniscus) on their right knees [34]. A sham operation was performed on the left knee of each operated mouse. Mice were sacrificed at specified time points after DMM and subjected to histological analyses [15, 17, 20]. To investigate the role of Prok2 in post-traumatic OA, mice that had undergone sham or DMM surgery were intra-articularly (IA) injected with 1 × 10^9^ pfu of control adenovirus (Ad-C) or adenovirus expressing murine Prok2 (Ad-Prok2). Alternatively, mice received IA injections of 10 μl/joint of 0.1% bovine serum albumin (BSA) in water or recombinant Prok2 (rProk2; 10 μg/10 μl in water) into their sham- or DMM-operated knees. To knock down Prok2 in joint tissues, mice received 1 × 10^9^ pfu of adenovirus expressing shRNA against Prok2 (Ad-shProk2). Adenovirus expressing scrambled shRNA (Ad-shControl) served as a control. All AI injections began 10 days after surgery and were repeated weekly for a total of three injections. All adenoviruses were purchased from Vector Biolabs (Malvern, PA), and rProk2 was obtained from PeproTech (Rocky Hill, NJ). Each group of mice was randomly assigned and sacrificed at specified time points after DMM for histological analysis. To examine the potential functions in joint tissues alone, mice received direct IA injection of 1 × 10^9^ pfu of Ad-Prok2 once a week for 3 weeks. IA injection of Ad-C or Ad-HIF-2α were used as negative and positive controls, respectively. Mice were sacrificed at either 6 or 9 weeks after the first IA injection for histological analyses [15, 17, 20].

### von Frey test

Sham- or DMM-operated mice received IA injection of 1 × 10^9^ pfu of Ad-C or Ad-Prok2 once a week for 3 weeks. The von Frey assay was conducted to assess hindpaw mechanical allodynia, following established procedures [17, 31, 35]. Briefly, behavior tests were conducted at specified time points after sham or DMM surgery. Mice were placed individually in small cages with a mesh floor and allowed to acclimatize to the test environment for at least 15 min. When the mouse ceased exploratory behavior, a von Frey filament was applied perpendicularly to the plantar surface of the paw until the filament buckled; it was then held there for a maximum of 3 s. A positive response was recorded if the paw was sharply withdrawn upon filament pressure or flinching was observed upon removal of the filament. Mechanical force was determined using a simplified up-down method, and mice were randomly assigned for testing by two blinded observers [35].

### Histological analysis

Mouse knee joint tissues and human OA cartilage were fixed in 4% paraformaldehyde, decalcified in 0.5M EDTA (pH 8.0), embedded in paraffin, and sectioned at 5-μm thickness [15, 17]. Sections were deparaffinized in xylene, hydrated with graded ethanol, and stained with safranin-O and hematoxylin. Safranin-O staining images were acquired using an Aperio CS2 slide scanner (Leica Biosystems, Richmond, IL). For the evaluation of OA severity, we used three different sections selected at approximately 100-μm intervals. Osteoarthritis Research Society International (OARSI) grade scores (ranging from 0 to 6) and osteophyte maturity (ranging from 0 to 3) were measured. The OARSI grade was expressed as the maximum score observed among the medial femoral condyle, medial tibial plateau, lateral femoral condyle, and lateral tibial plateau [36]. Osteophyte maturity was scored according to previously established criteria [15, 17]. Each section was scored independently by three blinded observers, and the results are presented as the average values obtained.

### Mouse cartilage explant culture

Cartilage explants were obtained from the femurs of 4-day-old ICR mice and cultured in complete medium. The explants were treated with 1 × 10^9^ pfu of Ad-Prok2 for 24 h. The same dosages of Ad-C and Ad-HIF-2α were used as negative and positive controls, respectively [11]. Alternatively, explants were treated with 100 ng/ml of rProk2 for 24 h. PBS and 100 ng/ml of IL-6 were applied as negative and positive controls, respectively [12]. Subsequently, the explants were fixed with paraformaldehyde, embedded in paraffin, sectioned at thickness of 5-um, and visualized by immunofluorescence microscopy, as described below.

### Immunohistochemistry (IHC) and immunofluorescence microscopy

Immunostaining of Prok2 in human and mouse joint sections was performed using the Dako LSAB2 horseradish peroxidase kit (Agilent, Santa Clara, CA). Briefly, slide sections were incubated overnight at 4℃ with a rabbit anti-Prok2 antibody (Abcam, Cambridge, MA; ab76747, 1:1000 dilution). The sections were further incubated with Dako Envision^+^ System HRP-labeled polymer reagents, and immunoreactive proteins were visualized using the Dako AEC high-sensitivity substrate chromogen solution (Agilent). Images were acquired using an Aperio CS2 slide scanner. The IHC images of Prok2 were semi-quantified using the Positive Pixel Count version 9 of the Aperio ImageScope Viewer software. All three staining intensity ranges (weak, positive, and strong) were considered as positive. The percentage of positive pixels was calculated relative to the total number of pixels in the sections [31]. For immunofluorescence microscopy, explants sections were incubated overnight at 4℃ with an anti-type II collagen antibody (Sigma, St. Louis, MO; MAB8887, 1:350 dilution) and an anti-MMP13 antibody (Abcam; ab51072, 1:250 dilution), and then with AlexaFluor 488 goat anti-mouse IgG (Thermo Fisher Scientific, Waltham, MA; A11029, 1:500 dilution) or AlexaFluor 594 goat anti-Rabbit IgG (Thermo Fisher Scientific; A11037, 1:500 dilution). Fluorescence images were obtained using a Fluoview FV 1000 confocal laser-scanning microscope (Zeiss, Oberkochen, Germany).

### Primary culture of mouse articular chondrocytes

Mouse articular chondrocytes were isolated from the femoral condyles and tibial plateaus of 4-day-old ICR mouse pups by 0.2% collagenase digestion method [37, 38]. Pooled chondrocytes were then plated at 3 × 10^5^ cells per 35-mm dish in Dulbecco’s modified Eagle’s medium (DMEM; Gibco, Waltham, MA) supplemented with 10% fetal bovine serum and antibiotics. The cells were treated with indicated concentration of rProk2 or infected with the specified multiplicity of infection (MOI) of control adenovirus (Ad-C) or adenovirus expressing Prok2 (Ad-Prok2), HIF-2α (Ad-HIF-2α) [11], or ZIP8 (Ad-ZIP8) [15]. All viruses were obtained from Vector Biolabs.

### Reverse transcription-polymerase chain reaction (RT-PCR) and quantitative RT-PCR (qRT-PCR) analyses

Total RNA was extracted from mouse chondrocytes using the TRI reagent (Molecular Research Center Inc., Cincinnati, OH), reverse transcribed, and the resulting cDNA was amplified by PCR. The sequences of the PCR primers for ADAMTS5, aggrecan, type II collagen, GAPDH, HIF-2α, and SOX9 were as previously described [15, 17, 20]. Additionally, the following primer sequences were used: 5′-TGCATATCTTCATCATGCTCCT-3′ (sense) and 5′-GTTTCCTCACGAAGGGGATCTT-3′ (antisense) for Prok1; 5′-CTCGGAAAGTTCCATTTTGG-3′ (sense) and 5′-TTCCGGGCCAAGCAAATAAACC-3′ (antisense) for Prok2; 5′-CAGCGCACATGAAGACTTG-3′ (sense) and 5′-GTCATCTTCGGTTTCCTGAG-3′ (antisense) for ProkR1; and 5′-GAACTCCACGTGAGCGCA-3′ (sense) and 5′-GGGCATGTTGATGATGC-3′ (antisense) for ProkR2. qRT-PCR was performed in a CFX Connect Real-Time PCR Detection System (Bio-Rad, Hercules, CA) using SYBR Premix Ex Taq (TaKaRa Bio Inc., Shiga, Japan). The reactive gene expression levels were analyzed using the 2^−ddCt^ method.

### Microarray analysis

Microarray data obtained from mouse articular chondrocytes, which were either treated with IL-1β, overexpressing HIF-2α via Ad-HIF-2α infection, or overexpressing ZIP8 via Ad-ZIP8 infection, have been previously deposited in the Gene Expression Omnibus under accession codes GSE104793 (IL-1β), GSE104794 (HIF-2α), and GSE104795 (ZIP8). The detailed methods for generating these microarray data were reported in a previous publication [17].

### Enzyme-linked immunosorbent assay (ELISA)

Human sera were collected from individuals classified as either KL grade 0 (non-OA) or KL grade 3 or 4 (OA) patients within the population-based Hallym Aging Study cohort [32, 33]. Mouse sera were collected at two time points: 8 weeks after sham or DMM surgery and 3 weeks after IA injection of 1 × 10^9^ pfu of Ad-C (control) or Ad-HIF-2α. In these experiments, both knees of each mouse underwent sham/DMM surgery or IA injections. Serum aliquots were stored at −80°C until analysis. The levels of circulating Prok2 in human or mouse sera were determined using human- or mouse-specific ELISA kits for Prok2 (MyBioSource, San Diego, CA; MBS90962 and MBS938170). Additionally, the levels of Prok2 secreted by primary-culture mouse articular chondrocytes were determined using a mouse-specific ELISA kit (MyBioSource) following the manufacturer’s protocol. Culture media (5 ml) from 1.5 × 10^6^ cells (five 35-mm dishes) infected with Ad-C, Ad-HIF-2α, or Ad-Prok2 were concentrated to 100 μl using Amicon ultra centrifugal filters with a nominal molecular weight limit of 5 kDa (Sigma).

### Statistical analysis

Statistical analyses were conducted using IBM SPSS version 28.0 software, with a significance level at 0.05. Normality of the data and homogeneity of variance were assessed using the Shapiro-Wilk and Levene’s test, respectively. Parametric data were compared using paired *t*-test for qRT-PCR data and IHC quantification, and Student’s *t* tests were applied to the results of the von Frey test. Non-parametric data based on the ordinal grading systems (OARSI grade and osteophyte maturity) were compared using the Mann-Whitney *U* test for two groups or Kruskal-Wallis with post hoc Bonferroni test for multi-groups. Parametric data are presented as mean ± 95% CI (confidence interval) or s.e.m with accompanying *P*-values. Non-parametric data are presented as median ± interquartile range (IQR) with *P*-values.

## Results

### Prok2 is upregulated in OA chondrocytes as a target of HIF-2α

**Fig. 1:**
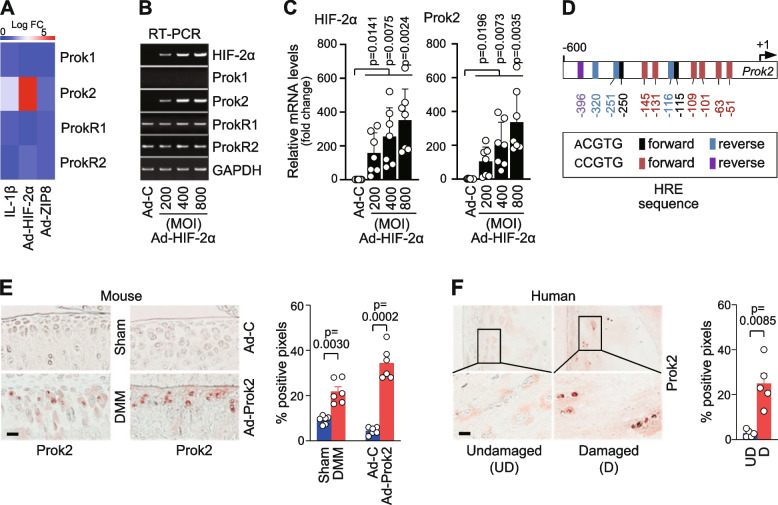
Prok2 is a target of HIF-2α in chondrocytes and exhibits upregulation in OA cartilage of mice and humans. **A** Primary-culture mouse chondrocytes were treated with vehicle alone or IL-1β (1 ng/ml, 36 h). Alternatively, chondrocytes were infected with 800 MOI of Ad-C (control), Ad-HIF-2α, or Ad-ZIP8 for 36 h. Presented is a heatmap of microarray analysis data for prokineticins (Prok) and their receptors (ProkR, *n*=3). **B**, **C** RT-PCR (**B**) and qRT-PCR (**C**) analysis of the indicated molecules in chondrocytes infected with 800 MOI of Ad-C (control) or the indicated MOI of Ad-HIF-2α for 36 h (*n*=7). **D** Positioning of HRE sequences in *Prok2* promoter. **E** Representative immunostaining images of Prok2 in cartilage from sham- or DMM-operated mice and semi-quantification of Prok2 positive pixels (*n*=6 mice per group). **F** Immunostaining images and semi-quantification of Prok2 positive pixels in damaged and undamaged human OA cartilage (*n*=5 patients). Values are presented as mean ± 95% CI, and significance was evaluated by paired *t*-test. Scale bar: 50 μm

We initially screened the mRNA levels of prokineticins and their receptors using microarray data obtained from OA-like chondrocytes. These chondrocytes were generated by treating primary-culture mouse chondrocytes with the pro-inflammatory cytokine IL-1β [10], or by infecting them with adenovirus expressing critical cellular mediators of OA pathogenesis, such as HIF-2α (Ad-HIF-2α) [11] or ZIP8 (Ad-ZIP8) [15]. Our microarray data analysis revealed that the mRNA level of Prok2 was significantly increased in chondrocytes overexpressing HIF-2α, while the mRNA levels of other prokineticins and their receptors remained unaltered (Fig. 1A). We further confirmed this finding through RT-PCR (Fig. 1B) and qRT-PCR (Fig. 1C) analyses, which demonstrated a significant elevation in Prok2 mRNA levels upon HIF-2α overexpression. Additionally, our analysis of the *Prok2* promoter revealed the presence of multiple hypoxia-response element (HRE) sequences, including five ACGTG and seven CCGTG motifs, within positions of −600 to +1 (Fig. 1D). This suggests that Prok2 is a direct target of HIF-2α in chondrocytes. Given the crucial role of HIF-2α as a catabolic regulator of OA pathogenesis [11, 15, 38], we sought to explore the potential functions of Prok2 in OA by investigating its expression in OA cartilage of both mice and humans. Immunostaining and semi-quantitation analysis revealed a significant increase in Prok2 protein levels in post-traumatic mouse OA cartilage induced by DMM surgery (Fig. 1E). Similarly, Prok2 protein levels were significantly higher in damaged human OA cartilage compared to the corresponding undamaged cartilage tissue (Fig. 1F). These findings collectively suggest that Prok2 may play a role in OA pathogenesis.

### Prok2 modulates the expression levels of matrix-degrading enzymes in chondrocytes

**Fig. 2:**
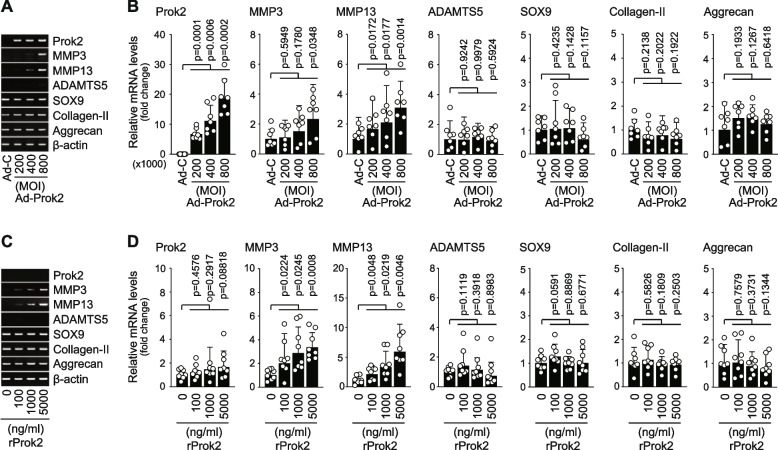
Prok2 stimulates expression of MMP3 and MMP13 in chondrocytes. **A**, **B** RT-PCR (**A**) and qRT-PCR (**B**) of the indicated molecules in chondrocytes infected with 800 MOI of Ad-C or the indicated MOI of Ad-Prok2 for 36 h (*n*=7 cell cultures). **C**, **D** RT-PCR (**C**) and qRT-PCR (**D**) of the indicated molecules in chondrocytes treated with vehicle alone or the indicated amount of recombinant Prok2 (rProk2) for 36 h (*n*=8 cell cultures). Values are presented as mean ± 95% CI, and significance was evaluated by paired *t*-test

To investigate the potential role of Prok2 in OA cartilage destruction, we conducted experiments to determine if Prok2 has an effect on the expression of matrix-degrading enzymes and cartilage extracellular matrix (ECM) molecules in chondrocytes. To do this, we overexpressed Prok2 in primary-culture chondrocytes using Ad-Prok2 infection and evaluated the expression levels of key matrix-degrading enzymes, including MMP3, MMP13, and ADAMTS5, which play pivotal roles in cartilage degradation during OA pathogenesis [7–9]. Our results from RT-PCR and qRT-PCR analyses revealed that adenoviral overexpression of Prok2 led to an upregulation of MMP3 and MMP13. However, this overexpression did not significantly alter the expression levels of ADAMTS5, SOX9, type II collagen, or aggrecan (Fig. 2A, B). Similarly, when we applied recombinant Prok2 (rProk2), it also resulted in an upregulation of MMP3 and MMP13 without significantly affecting the expression levels of ADAMTS5, SOX9, type II collagen, or aggrecan (Fig. 2C, D).

**Fig. 3:**
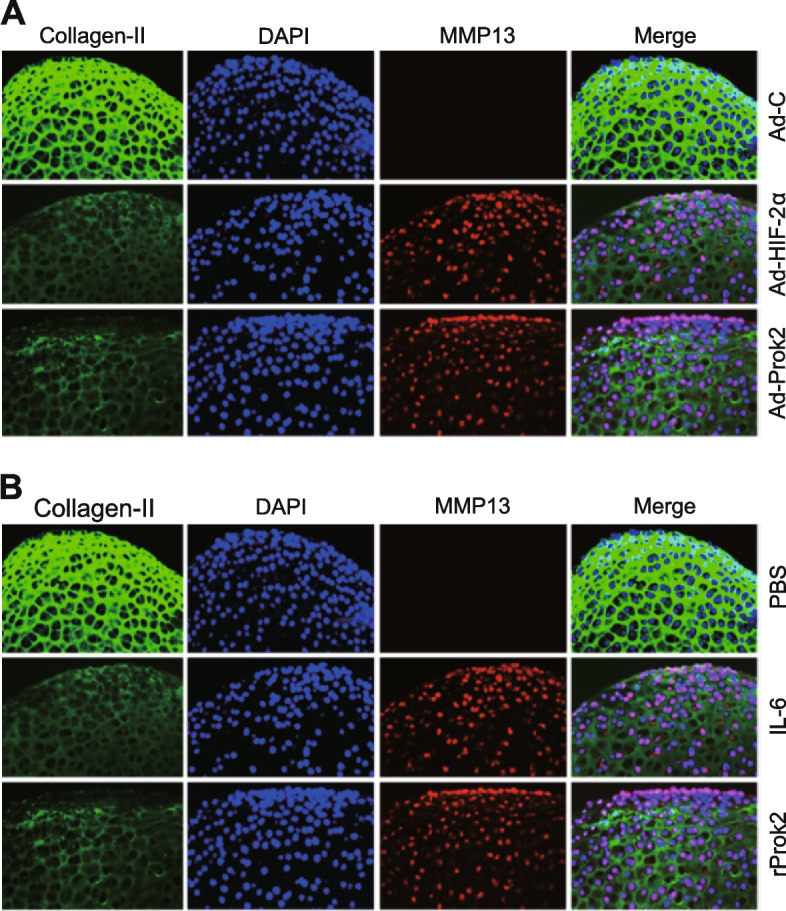
Prok2 modulates protein levels of MMP13 and type II collagen in cartilage explants. **A** Mouse cartilage explants, sourced from 4-day-old ICR mice, were treated with 1 × 10^9^ pfu of Ad-C, Ad-HIF-2α, or Ad-Prok2 for 24 h. Presented are representative immunofluorescence images of type II collagen, MMP13, and DAPI in sections of cartilage explants (*n*=10 explants). **B** Mouse cartilage explants were treated with PBS as a control, 100 ng/ml of IL-6 as a positive control, and 100 ng/ml of rProk2 for 24 h. Representative immunofluorescence images of type II collagen, MMP13, and DAPI in sections of cartilage explants are presented (*n*=10 explants)

We further confirmed the impact of Prok2 on the expression levels of MMP13 in cartilage explant cultures. As shown in Fig. 3A, consistent with a previous findings [12], adenoviral overexpression of HIF-2α markedly increased the protein level of MMP13 (Fig. 3A). Immunofluorescence microscopy revealed that Prok2 overexpression, similar to the effects of HIF-2α overexpression, led to an increase in the protein expression level of MMP13 in cartilage explants (Fig. 3A). Additionally, treatment of cartilage explants with rProk2 also resulted in an upregulation of MMP13 at the protein level in the explants (Fig. 3B). Notably, the effects of rProk2 were comparable to those observed following treatment with IL-6, which has previously been shown to regulate the expression of MMP13 in cartilage explants [12]. Alongside the increase in MMP13 protein levels, Prok2 overexpression (Fig. 3A) and rProk2 treatment (Fig. 3B) both caused a downregulation of type II collagen, which is a substrate of MMP13, at the protein level in cartilage explants (Fig. 3B). Importantly, these effects were observed without any detectable alterations in the mRNA levels of type II collagen (Fig. 2B). Taken together, our results suggest that the upregulation of the matrix-degrading enzyme MMP13 leads to the degradation of type II collagen, with no significant impact on its mRNA levels.

### The overexpression of Prok2 in joint tissues exacerbates post-traumatic OA cartilage destruction and mechanical allodynia in mice

**Fig. 4:**
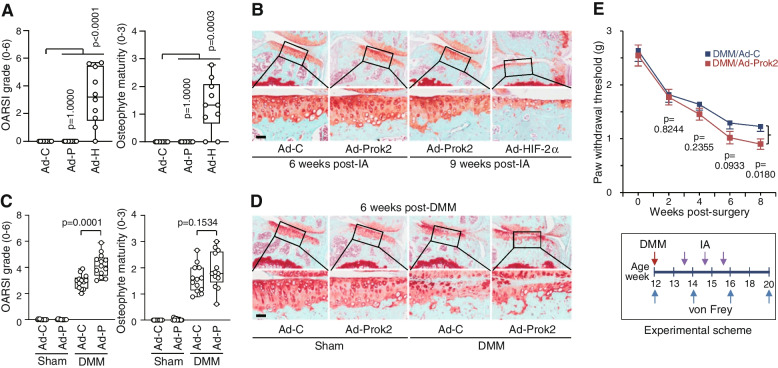
Adenoviral overexpression of Prok2 in joint tissues exacerbates post-traumatic OA cartilage destruction and hindpaw mechanical allodynia in mice. **A**, **B** Mice were IA injected with 1 × 10^9^ pfu of Ad-C (control), Ad-Prok2 (Ad-P), or Ad-HIF-2α (Ad-H) once a week for 3 weeks (*n*=10 mice per group). OARSI grade and osteophyte maturity were determined at 9 weeks after the first IA injection (**A**). Representative safranin-O staining images of joint sections at 6 weeks and 9 weeks after the first IA injection (**B**). **C**, **D** Sham- or DMM-operated mice were IA injected with 1 × 10^9^ pfu of Ad-C (control) or Ad-Prok2 (Ad-P) once a week for 3 weeks (*n*=13 mice per group). OARSI grade and osteophyte maturity were determined at 6 weeks after surgery (**C**). Representative safranin-O staining images of joint sections at 6 weeks after surgery (**D**). **E** von Frey assays were performed at the indicated weeks after DMM operation (*n*=22 mice per group). Data for OARSI grade and osteophyte maturity are presented as median ± interquartile range (IQR) and paw withdrawal threshold is presented as mean with s.e.m. Significance was evaluated by Kruskal-Wallis with post hoc Bonferroni (**A**), Mann-Whitney *U* test (**B**), and Student’s *t* test (**E**), respectively. Scale bar: 50μm

The potential functions of Prok2 in OA development were investigated by overexpressing it in mouse knee joint tissues through IA injection of Ad-Prok2. An adenoviral system was utilized to efficiently deliver the Prok2 transgene into joint tissues, as previously described [15, 17]. While the adenoviral overexpression of HIF-2α, a positive control [11, 13], led to cartilage erosion, the exclusive overexpression of Prok2 alone did not induce any OA-like changes, including cartilage destruction and osteophyte formation, at both 6 and 9 weeks after the initial IA injection of Ad-Prok2 (Fig. 4A, B). However, when examining the effects of Prok2 overexpression in knee joint tissues under DMM-induced post-traumatic OA, it was found that adenoviral overexpression of Prok2 in joint tissues of DMM-operated mice significantly exacerbated cartilage destruction, though it did not significantly affect osteophyte maturity (Fig. 4C, D). Compared to Ad-C treatment, IA injection of Ad-Prok2 increased the median OARSI grade at 6 weeks after DMM surgery from 3.00 (IQR 2.44–3.11) to 4.11 (IQR 3.56–4.61, *p*=0.0001), indicating a worsening of cartilage damage.

While the function of Prok2 in OA-associated mechanical allodynia was previously unknown, several studies have demonstrated its significant role in nociception [39]. Notably, Prok2-deficient mice exhibited higher thresholds for thermal and mechanical stimuli compared to WT mice [40], Prok2 was also found to be upregulated in the peripheral nervous system in a chronic constriction injury-induced model [41]. Additionally, intraperitoneal injection of rProk2 in rodents induced hyperalgesia to noxious stimuli [42]. Therefore, we conducted an examination to determine whether Prok2 regulates mechanical sensitivity during the progression of OA. Using the von Frey assay in DMM-operated mice, we observed that adenoviral overexpression of Prok2 significantly increased their sensitivity to mechanical stimuli during OA progression (Fig. 4E). Specifically, compared to the control condition, Prok2 overexpression in DMM-operated mouse knee joints reduced the mechanical threshold from 1.23 ± 0.09 g to 0.90 ± 0.10 g (*p*=0.0180) at 8 weeks after DMM surgery. Prok2 overexpression in sham-operated mice did not yield any difference in the paw withdrawal threshold (data not shown). These findings collectively indicate that Prok2 overexpression exacerbates DMM-induced OA cartilage destruction and mechanical allodynia in mice.

**Fig. 5:**
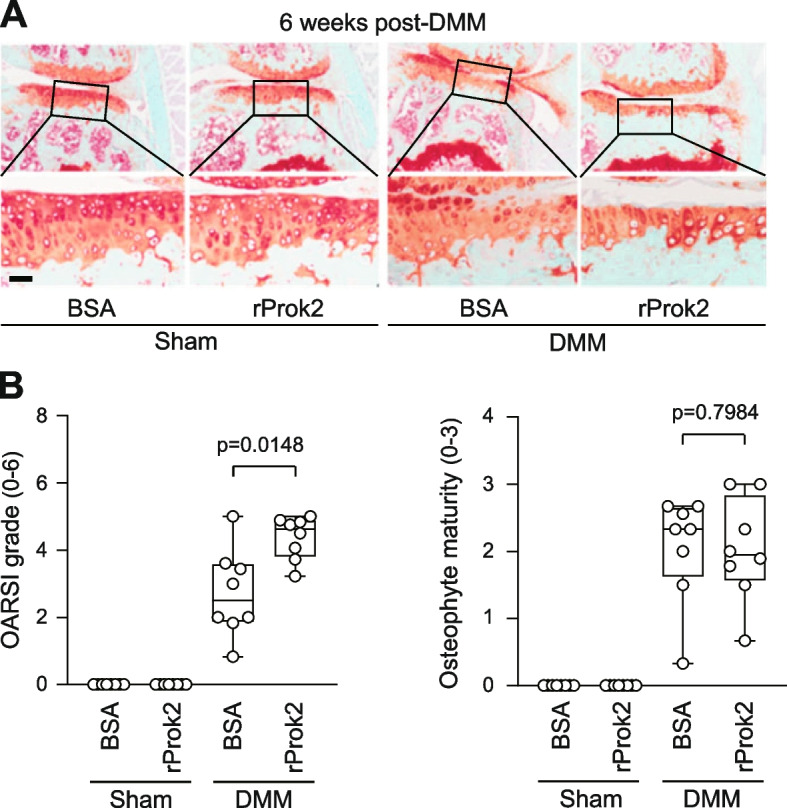
IA injection of recombinant Prok2 (rProk2) exacerbates post-traumatic OA cartilage destruction. **A**, **B** Sham- or DMM-operated mice were IA injected with BSA or rProk2 (10 μg/10 μ1/joint) once a week for 3 weeks (*n*=8 mice per group). Representative safranin-O staining images of joint sections at 6 weeks after surgery (**A**). Quantification of OARSI grade and osteophyte maturity at 6 weeks after surgery (**B**). Values are presented as median ± interquartile range (IQR) and significance was evaluated by Mann-Whitney *U* test. Scale bar: 50 μm

We additionally investigated the impact of rProk2 on post-traumatic OA cartilage destruction. To do this, we administrated IA injections of BSA (0.1%, 10 μl) as a control or rProk2 (10 μg in 10 μl) to sham- or DMM-operated mice. IA injection of rProk2 into sham-operated knee joints did not induce any OA-like changes in the joint tissues at 6 weeks post-surgery (Fig. 5A, B). However, IA injection of rProk2 into DMM-operated knee joints significantly exacerbated cartilage destruction. In comparison to the vehicle-treated group, mice receiving IA injections of rProk2 exhibited a substantial increase in the median OARSI grade, from 2.50 (IQR 1.92–3.53) to 4.63 (IQR 3.89–4.86, *p*=0.0148), respectively (Fig. 5A, B). Notably, osteophyte formation remained unaffected by IA injection of rProk2 in DMM-operated mice (Fig. 5A, B). In conjunction with the data obtained from adenoviral overexpression of Prok2, these results collectively indicate that Prok2 plays a role in regulating OA cartilage destruction in DMM-operated mice, while osteophyte formation remains unaffected.

### Knockdown of Prok2 has no significant effect on DMM-induced cartilage destruction

**Fig. 6:**
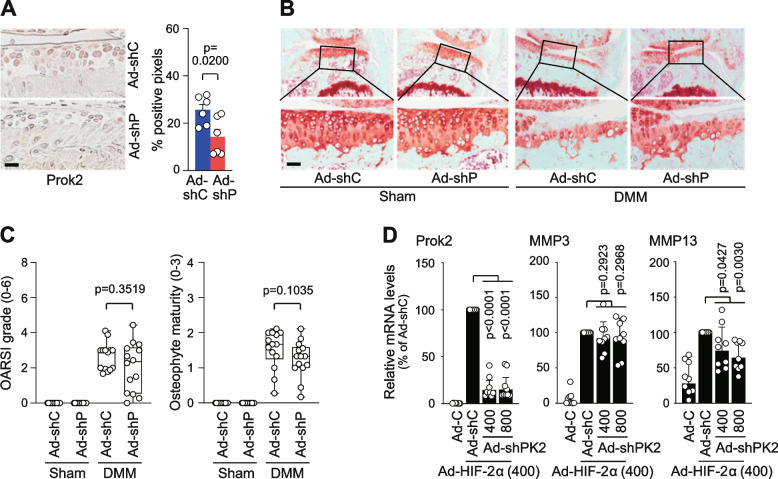
Knockdown of Prok2 in joint tissues alone is insufficient to inhibit post-traumatic OA cartilage destruction. **A** Representative immunostaining images of Prok2 (left panel) and semi-quantification of Prok2-positeive pixels (right panel) in cartilage sections of mice received IA injection of 1 × 10^9^ pfu of Ad-shC (control) or Ad-shProk2 (*n*=6 mice per group). **B**, **C** Sham and DMM-operated mice were subject to IA injections of 1 × 10^9^ pfu of Ad-shC or Ad-shProk2 once a week for 3 weeks. Representative safranin-O staining images (**B**) and scoring of OARSI grade and osteophyte maturity (**C**, *n*=14 mice per group). **D** qRT-PCR analysis of the indicated molecules in primary-culture chondrocytes treated with 400 MOI of Ad-C and Ad-HIF-2α, with or without the specified MOI of Ad-sh-C and Ad-shProk2 (*n*=9). Values are presented as mean ± 95% CI, and significance was evaluated by paired *t*-test

To further investigate the role of Prok2 in OA pathogenesis, we downregulated Prok2 in whole-joint tissues by administrating an adenovirus expressing shRNA against Prok2 (Ad-shProk2) via IA injection. This IA injection of Ad-shProk2 effectively reduced Prok2 protein levels in cartilage tissue (Fig. 6A). However, mice that received IA injections of 1 × 10^9^ pfu of Ad-shProk2 did not show significant modulation of OA cartilage destruction, though there was a trend toward inhibition (Fig 6B, C). Likewise, the maturity of osteophyte remained unaffected by the knockdown of Prok2 (Fig 6B, C). In line with its effects on cartilage destruction, the expression levels of MMP3 remained unaffected by Prok2 knockdown in primary-culture chondrocytes, whereas MMP13 expression was only reduced slightly at a high dosage of Ad-shProk2 (Fig. 6D). Our results collectively suggest that Prok2 knockdown alone is not sufficient to modulate post-traumatic OA cartilage destruction, while its overexpression exacerbates cartilage destruction.

### Prok2 is undetectable in the sera of mice with experimental OA or humans with OA

**Fig. 7:**
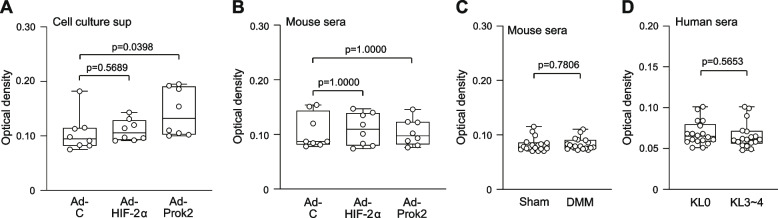
Prok2 is not detected in the sera of OA model mice or human OA patients. **A** Primary-culture mouse chondrocytes were infected with 800 MOI of Ad-C, Ad-HIF-2α, or Ad-Prok2 for 36 h. Five milliliters of cell culture supernatant from five 35-mm dishes was concentrated to 100 μl an ELISA was used to detect secreted Prok2 (*n*=8 independent cultures). **B** Sera were collected from mice IA injected in both knees with 1 × 10^9^ pfu of Ad-C, Ad-HIF-2α, or Ad-Prok2 once a week for 3 weeks. Prok2 levels in 100 μl of collected sera were determined by ELISA (*n*=8 mice per group). **C** Sera was collected from mice that were sham- or DMM-operated in both knees and sampled at 8 weeks after the operation. Prok2 levels were determined by ELISA from 100 μl of collected sera (*n*=18 mice per group). **D** Prok2 levels were determined from 100 μl of sera collected from KL grade 0 non-OA individuals and KL grade 3~4 OA patients (*n*=18 females per group). Because the Prok2 levels in all samples (except the Ad-Prok2-infected chondrocytes in A) were below the range of detection, the detected values are presented as optical density. Statistical values are presented as means ± s.e.m, and were assessed with paired *t*-test for ELISA of cell culture media, ANOVA with Bonferroni’s post hoc test for ELISA of mouse serum, and Student’s *t* test for ELISA of human serum

As Prok2 is a secreted protein that is upregulated in OA cartilage and plays a role in OA cartilage destruction, we investigated its potential as a biomarker for experimental OA in mice and human OA. Our initial step involved examining Prok2 protein levels secreted by primary-culture mouse chondrocytes that were overexpressing either HIF-2α or Prok2. Through ELISA, we were able to detect the secretion of Prok2 from Prok2-overexpressing chondrocytes. However, the levels Prok2 secreted by HIF-2α-overexpressing chondrocytes were below the detection limit of our ELISA (Fig. 7A). We also analyzed the presence of secreted Prok2 in sera (100 μl) of mice subjected to IA injection in both knees with 1 × 10^9^ pfu of Ad-C, Ad-HIF-2α, or Ad-Prok2 once a week for 3 weeks. Our ELISA results indicated that Prok2 levels were consistently below the lower limit of detection in mice that received IA injections of all these adenoviruses (Fig. 7B). Furthermore, we assessed secreted Prok2 levels in the sera of mice that underwent sham or DMM operation on both knees. Our ELISA analysis could not detect secreted Prok2 at 8 weeks post-surgery (Fig. 7C). Finally, we assessed serum Prok2 levels in randomly selected female individuals with KL grade 0 (non-OA) and KL grade 3 or 4 (OA) patients. Consistent with our findings in mouse experimental OA, the serum levels (100 μl) of Prok2 were undetectable in both non-OA individuals and KL grade 3 or 4 OA patients (Fig. 7D). These collective results suggest that serum Prok2 levels in both experimental OA mice and human OA patients are too low for Prok2 to be considered a viable candidate biomarker for OA.

## Discussion

Prokineticins (Prok1 and Prok2) constitute a novel class of chemokine-like peptides that bind to cognate G protein-coupled receptors (ProkR1 and ProkR2) to mediate a variety of biological functions [22–26]. In this study, we have demonstrated that Prok2 is upregulated in OA chondrocytes as a target of HIF-2α, contributing to cartilage destruction during OA pathogenesis. The catabolic capacity of Prok2 appears to manifest through the upregulation of MMP3 and MMP13 in chondrocytes, which are critical matrix-degrading enzymes associated with cartilage degeneration [7, 8]. Our previous study has shown that, during OA cartilage destruction, catabolic signaling of HIF-2α is significantly amplified by downstream signaling, further increasing the expression of matrix-degrading enzymes like MMP3 and MMP13 [11–17, 20]. Consequently, our current findings add Prok2 to the expanding list of HIF-2α-related catabolic signaling factors that are augmented during OA pathogenesis. While our study did not delve into the molecular mechanisms responsible for the upregulation of MMP3 and MMP13 by Prok2, it is known that various transcription factors regulate their expression levels. For instance, MTF1 and RORα have been independently identified as mediators of these matrix-degrading enzyme expressions downstream of ZIP8 and oxysterol metabolites, respectively [15, 17]. Therefore, it is plausible that the activation of as-yet-unidentified transcription factor(s) play a role in mediating the increased expression of MMP3 and MMP13 upon activation of prokineticin receptors by Prok2.

While the role of prokineticin signaling in OA pathogenesis has not been previously explored, its involvement in autoimmune diseases, such as RA, is relatively well-documented. A recent study reported elevated levels of Prok2 in the synovial fluid and plasma of RA patients, along with increased Prok2 and ProkR2 levels in CIA joint tissues. Furthermore, intraperitoneal administration of a prokineticin receptor antagonist was found to suppress the severity of arthritis [28]. Another study showed that blocking prokineticin receptors with a non-peptide ProkR1-preferring antagonist attenuated synovitis and joint destruction in the CIA model [29]. Our current findings represent the first evidence of prokineticin signaling pathway as a catabolic regulator of OA cartilage destruction. Among the components of the prokineticin signaling pathway, Prok2 appears to be the key player, as it was specifically upregulated in OA chondrocytes. However, it remains to be elucidated which prokineticin receptors are involved in mediating Prok2 signaling during OA cartilage destruction. Prokineticins have the capacity to bind to both ProkR1 and ProkR2 [21, 22], and our analysis indicated similar levels of expression in chondrocytes (Fig. 1B), suggesting that both receptors may contribute to Prok2 signaling in chondrocytes. In contrast to the exacerbation of DMM-induced OA cartilage destruction and upregulation of MMPs observed with adenoviral overexpression of Prok2, it is important to highlight that knockdown of Prok2 alone in joint tissues did not significantly affect DMM-induced post-traumatic OA cartilage destruction or HIF-2α-induced upregulations of MMPs. These results suggest that, while the overexpressed Prok2 appears to exert catabolic effects in OA, knocking down Prok2 is insufficient to inhibit HIF-2α-induced expression of the matrix-degrading enzymes and DMM-induced cartilage destruction. Consequently, it is likely that HIF-2α and DMM surgery induce the expression of matrix-degrading enzymes and cartilage destruction, respectively, through alternative pathways that are independent of Prok2 signaling.

In addition to exacerbating OA cartilage destruction, overexpressed Prok2 also intensified OA-associated mechanical allodynia in DMM-operated mice. These findings are consistent with previously reported functions of Prok2. For instance, Prok2 has been shown to be upregulated in the peripheral nervous system following chronic constriction injury [41]. Intraperitoneal injection of rProk2 in rodents induced hyperalgesia in response to noxious stimuli [42], and Prok2-deficient mice exhibited higher thresholds to thermal and mechanical stimuli compared to wild-type mice [40]. Prokineticin receptors are known to be highly expressed in nociceptor endings and dorsal root ganglia, and they provide tonic activation of TRPV1 and TRPA1, contributing to peripheral sensitization [39]. As such, we hypothesize that Prok2 may regulate peripheral sensitization through prokineticin receptors or nociceptors, such as TRPV1, in various OA joint tissues, including synovium, ligaments, and subchondral bone [31].

The observed upregulation of Prok2 in OA cartilage, along with its potential role as a catabolic regulator, raised the possibility of using secreted Prok2 as a diagnostic biomarker for OA. Indeed, many recent studies have suggested that circulating Prok2 in serum could serve as a biomarker for various diseases, including neurodegenerative diseases such as Alzheimer’s disease and Parkinson’s disease [43], low-grade gliomas [44], and Kawasaki disease (a self-limiting inflammatory disorder disease) [45]. However, our study revealed that the serum levels of Prok2 in experimental OA mice and human OA patients were consistently below the lower limit of detection for our ELISA. This suggests that, without more sensitive tools for detecting Prok2, circulating Prok2 would not be an effective diagnostic biomarker for OA. In contrast, our previous study indicated that secretory leukocyte peptidase inhibitor (SLPI) could be a potential diagnostic marker for OA [46]. Serum SLPI levels were found to be significantly increased before the onset of cartilage damage and remained elevated during DMM-induced cartilage destruction in mice. Additionally, SLPI protein levels showed marked increased in OA cartilage and the circulating blood of human OA patients. Therefore, in contrast to SLPI, Prok2 does not appear to be a suitable candidate for an OA biomarker.

## Conclusions

We have demonstrated that the upregulation of Prok2 in OA chondrocytes serves as a catabolic regulator, contributing to OA cartilage destruction in mice through the expression of matrix-degrading enzymes. However, it is important to note that knocking down Prok2 in joint tissues alone is insufficient to inhibit DMM-induced post-traumatic OA cartilage destruction. Prok2 is also implicated in OA-associated hindpaw mechanical allodynia. Despite its functional role in OA pathogenesis, the levels of Prok2 in the sera of both OA mice and humans are too low to consider circulating Prok2 as a potential biomarker for OA.

### Supplementary Information

**Additional file 1: Supplementary Figure 1.** Full, uncropped gel images.

## Data Availability

The data supporting the conclusions of this article are included within the article.

## Abbreviations

BSA: Bovine serum albumin
CI: Confidence of interval
CIA: Collagen-induced arthritis
DMM: Destabilization of the medial meniscus
ECM: Extracellular matrix
ELISA: Enzyme-linked immunosorbent assay
IA: Intra-articular
IL: Interleukin
HIF-2α: Hypoxia-inducible factor-2α
HRE: Hypoxia-response element
KL: Kellgren-Lawrence
MMP: Matrix metalloproteinase
MOI: Multiplicity of infection
OA: Osteoarthritis
Prok1: Prokineticin 1
PFU: Plaque forming unit
Prok2: Prokineticin 2
ProkR1: Prokineticin receptor 1
ProkR2: Prokineticin receptor 2
RA: Rheumatoid arthritis
(q)RT-PCR: (Quantitative) reverse transcription-polymerase chain reaction
RORα: Retinoid-related orphan receptor α
SLPI: Secretory leukocyte peptidase inhibitor
