# Supplementary figures and images for: Prokineticin 2 is a catabolic regulator of osteoarthritic cartilage destruction in mouse
Source: Arthritis Res Ther. 2023 Dec 6;25:236. doi: 10.1186/s13075-023-03206-4 (PMC10699050; doi:10.1186/s13075-023-03206-4)

Supplementary Figure 1. Full, uncropped gel images

Figure 1B

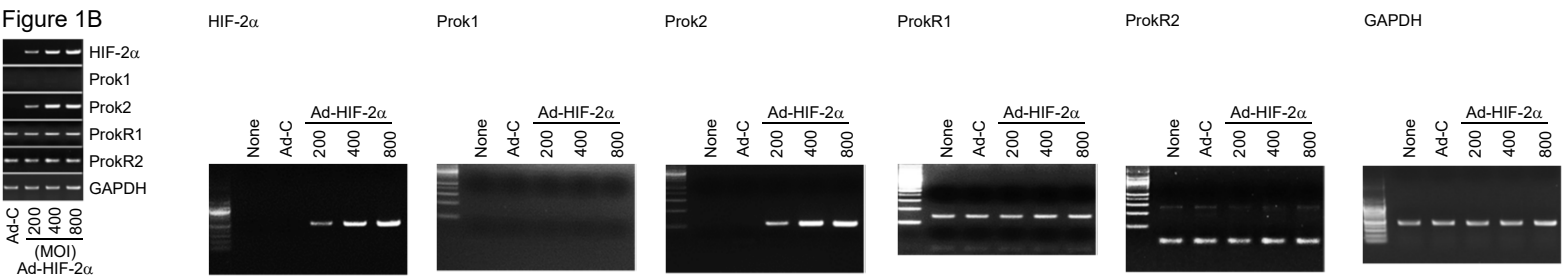

Figure 2A

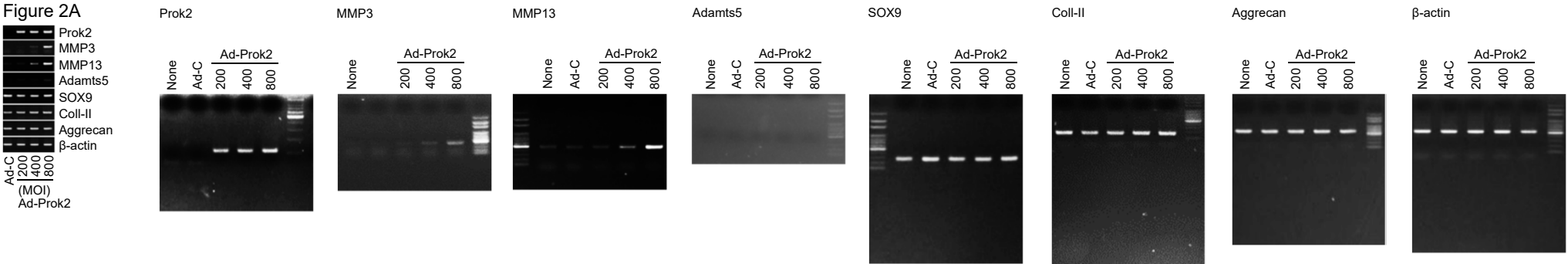

Figure 2C

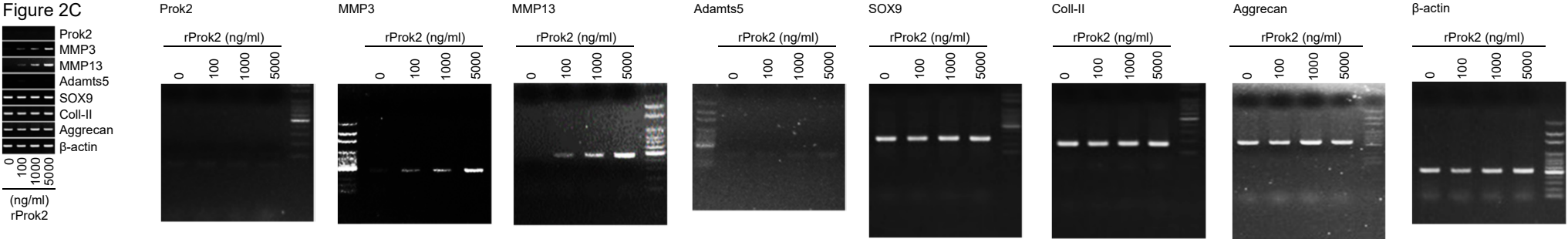

Supplement: Supplementary file 1 — Additional file 1: Supplementary Figure 1. Full, uncropped gel images. [file 13075_2023_3206_MOESM1_ESM.pdf]
